# Transport and kinase activities of CbrA of *Pseudomonas putida* KT2440

**DOI:** 10.1038/s41598-020-62337-9

**Published:** 2020-03-25

**Authors:** Larissa Wirtz, Michelle Eder, Kerstin Schipper, Stefanie Rohrer, Heinrich Jung

**Affiliations:** 1 Division of Microbiology, Department of Biology 1, Ludwig Maximilians University Munich, D-82152 Martinsried, Germany; 2 Present Address: Institute of Microbiology, Department of Biology, Heinrich-Heine-University, D-40225 Düsseldorf, Germany; 3 Present Address: Technical University of Munich, D-80333 Munich, Germany

**Keywords:** Kinases, Membrane proteins, Protein purification, Bacteriology

## Abstract

The CbrA/CbrB system is a two-component signal transduction system known to participate in the regulation of the cellular carbon/nitrogen balance and to play a central role in carbon catabolite repression in *Pseudomonas* species. CbrA is composed of a domain with similarity to proteins of the solute/sodium symporter family (SLC5) and domains typically found in bacterial sensor kinases. Here, the functional properties of the sensor kinase CbrA and its domains are analyzed at the molecular level using the system of the soil bacterium *P. putida* KT2440 as a model. It is demonstrated that CbrA can bind and transport L-histidine. Transport is specific for L-histidine and probably driven by an electrochemical proton gradient. The kinase domain is not required for L-histidine uptake by the SLC5 domain of CbrA, and has no significant impact on transport kinetics. Furthermore, it is shown that the histidine kinase can autophosphorylate and transfer the phosphoryl group to the response regulator CbrB. The SLC5 domain is not essential for these activities but appears to modulate the autokinase activity. A phosphatase activity of CbrA is not detected. None of the activities is significantly affected by L-histidine. The results demonstrate that CbrA functions as a L-histidine transporter and sensor kinase.

## Introduction

Transporters integral to cytoplasmic membranes usually catalyze the selective uptake of nutrients or the extrusion of metabolic end products and toxic solutes. However, some of these transporters play a central role also in signal transduction^1,2^. In bacteria, so-called trigger transporters (temporarily) interact with membrane components of signal transduction systems and modulate their activity^2^. For example, the lysine transporter LysP allows activation of a CadC-dependent acid stress response only when lysine can be taken up from the environment^3^. The C_4_-dicarboxylite transporter DcuB and the glucose-6-phosphate transporter UhpC interact with histidine kinases of specific two-component systems (TCSs) and stimulate phosphotransfer to the cognate response regulators when the respective substrate is present^1,4^.

While the interaction of transporters with separate signal transductions systems and the functional consequences are relatively well understood, little is known about the role of transporters that are covalently linked to domains typically found in bacterial signaling cascades. Prominent examples are members of the solute/sodium symporter family (SLC5)^5,6^. Besides sodium-motive force-dependent transporters for proline (PutP of archaea and bacteria^7^), monosaccharides (SGLT of bacteria and higher eukaryotes^8^) and others^9–11^, the family contains bacterial proteins in which a complete SLC5 domain is connected via a STAC (SLC5 and TCS Associated Component) domain to domains found in histidine kinases or diguanylate cyclase^5,12,13^. SLC5 transporters fused via STAC to histidine kinase domains are usually associated with response regulators and resemble TCSs. CbrA/CbrB represents such a histidine kinase/response regulator pair^14–16^. The TCS functions as a global metabolic regulator that impacts virulence, biofilm formation, and antibiotic resistance of *Pseudomonas* species^16,17^. More specifically, CbrA/CbrB regulates carbon utilization and together with NtrB/NtrC ensures a balanced carbon/nitrogen relationship^16,18^. Thereby, CbrB can directly regulate expression of different σ^N^ dependent catabolic pathways, *e.g*., the *histidine utilization* (*hut*) operon^18,19^. In addition, CbrA/CbrB is involved in carbon catabolite repression (CCR)^15,20–22^. In the presence of less-favorable substrates (*e.g*., L-histidine), the phosphorylation cascade of CbrA/CbrB is activated leading to the expression of the small RNAs *crcZ* and *crcY* that in turn bind the CCR protein Crc resulting in an increased translation of Crc target mRNAs^23,24^.

**Figure 1:**
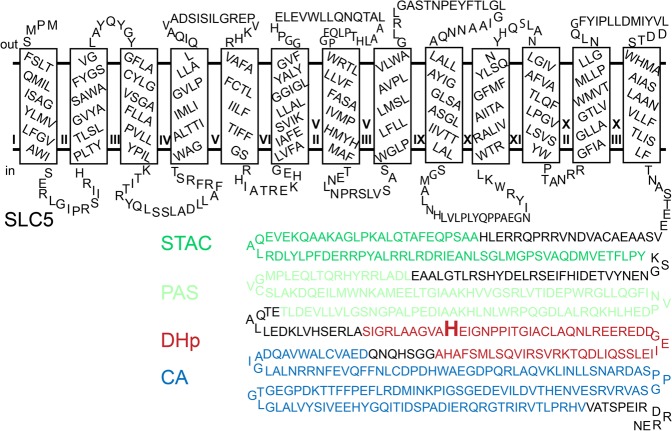
Topology model of CbrA of *P. putida* KT2440 showing the domains predicted by Sepulveda and Lupas (2017)^13^. The SLC5 domain (black) entails 13 TMDs connected by hydrophilic loops, a STAC domain (green) connects the SLC5 domain to the cytosolic domain and is followed by the domains PAS (light green), DHp (red) and CA (blue). The predicted phosphorylation site (His766) in the DHp domain is highlighted.

Similar to other bacterial members of the SLC5 family (*e.g*., the proline/sodium symporter PutP^5^), the SLC5 domain of CbrA (about 500 amino acids) consists of 13 transmembrane domains (TMDs) that are connected by short hydrophilic loops (Fig. 1). The cytoplasmic C terminus of the SLC5 domain is covalently linked to the above mentioned STAC domain (72 amino acids). The STAC domain is suggested to mediate interactions with other proteins or control the transport cycle of the SLC5 domain^12^. It follows a PAS domain (about 115 amino acids) that may bind a yet to be identified signal molecule, a dimerization and histidine phosphotransfer domain (DHp, about 65 amino acids), and a catalytic ATP-binding domain CA (about 115 amino acids)^13^. The individual domains are connected by linker sequences (Fig. 1). Upstream of *cbrA* and partially overlapping with the gene, an ORF encoding a small hydrophobic peptide termed CbrX (58 amino acids) is located^25^. Translation of *cbrX* and *cbrA* are coupled, thereby the amino acid sequence of CbrX seems to be unimportant for the stability and function of CbrA^14^.

A mutant of *Pseudomonas fluorescens* SBW25 devoid of known histidine uptake systems was previously shown to grow on L-histidine, and CbrA was identified as being responsible for L-histidine uptake in that strain^26^. These results suggest that CbrA responds to extracellular L-histidine, and that transport and signal transduction are coupled^26^. Later investigations revealed, however, that the histidine kinase can function independently from the SLC5 domain and more likely relies on a not yet identified intracellular signal^13,14^.

Here, we set out to further explore the functional properties and interactions of the individual domains of CbrA in the soil bacterium *Pseudomonas putida* KT2440. For this purpose, we deleted individual domains of CbrA and analyzed the impact of the deletion on the expression of known target genes in *P. putida* KT2440. In addition, we genetically engineered, expressed, and purified individual domains and truncated versions of CbrA and compared the functional properties with wild type CbrA. We demonstrated that the SLC5 domain of CbrA transports L-histidine, and analyzed transport kinetics, substrate specificity, and driving force of the transport process. Furthermore, using ^32^P-ATP, we show that CbrA and CbrAΔSLC5 can autophosphorylate at His766 and transfer the phosphoryl group to CbrB. The SLC5 domain is not essential for these activities but appears to modulate the autokinase activity. A phosphatase activity of CbrA or CbrAΔSLC5 leading to dephosphorylation of CbrB~^32^P was not detected. Although CbrA can transport L-histidine, autokinase, phosphotransfer and phosphatase activities are not influenced by the amino acid.

## Results

### The SLC5 domain of CbrA of *P. putida* KT2440 transports L-histidine

**Figure 2:**
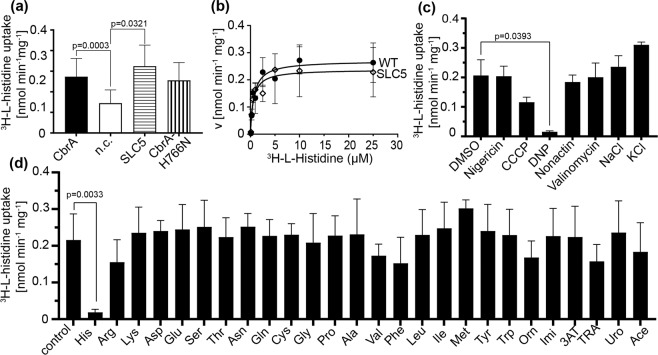
Properties of the CbrA-dependent transport of L-histidine. (**a**) Initial rates of ^3^H-L-histidine uptake into cells of *P. putida* LW1 (Δ*cbrA* Δ*hutTH* Δ*hutWX*) harboring pUCP-Tc (n.c.), pUCP-Tc-*cbrA*, or pUCP-Tc with given *cbrA* variants. Cells were suspended in 100 mM Tris/MES buffer, pH6.0 (OD_600_ = 5), and transport was initiated by addition of ^3^H-L-histidine at a final concentration of 1 µM. (**b**) Michaelis-Menten kinetics of ^3^H-L-histidine uptake by CbrA and the SLC5 domain of CbrA. Transport was measured as described in (**a**) with substrate concentrations ranging from 0.1 to 25 µM. The initial rate of transport determined at each substrate concentration was corrected for background activity (rate without CbrA or SLC5 domain). Data were fitted and kinetic parameters were determined using GraphPad Prism. (**c**) Effect of ionophores, NaCl, and KCl on the initial rate of ^3^H-L-histidine uptake in cells transformed with pUCP-Tc-*cbrA*. The activity of cells in the presence of the solvent DMSO served as reference for all measurements with ionophores. Activities in the presence of NaCl and KCl were compared with the activities of CbrA shown in (**a**). (**d**) Analysis of the substrate specificity of CbrA. ^3^H-L-histidine uptake by CbrA was measured as described in (**a**) without additions (control) and in the presence of 100 µM (100fold molar excess) of proteinogenic amino acids, ornithine (Orn), imidazole (Imi), 3-amino-1,2,4-triazole (3AT), 1,2,4-triazolyl-3-alanine (TRA), urocanate (Uro), or acetate (Ace). For all experiments, standard deviations were calculated from minimum three independent experiments. Welch’s t-test was applied for statistical analyses.

Based on the previous observation that CbrA of *P. fluorescens* SBW25 transports histidine^26^, we set out to test if this is also the case for CbrA of *P. putida* KT2440 and, if so, to further characterize the transport process. For this purpose we generated a strain devoid of *cbrA* and other genes for putative histidine transporters [*hutT* (PP_5031, encodes the main inducible histidine transporter) and *hutXW* (PP_3558/PP_3559, encode a putative periplasmic binding protein and an integral membrane component of a putative ABC transporter)^19^. In addition, *hutH* (PP_5032) encoding the enzyme for the first step of histidine degradation^19^ was deleted. Gene deletions were generated by homologous recombination using the pNPTS138-R6KT suicide vector^27^. The resulting mutant *P. putida* LW1 was transformed with pUCP-Tc-*cbrA* or pUCP-Tc (negative control), *cbrA* expression from the *lac* promoter was induced by IPTG, and uptake of ^3^H-L-histidine was analyzed (Fig. 2a). While the strain without *cbrA* transported ^3^H-L-histidine with an initial rate of 0.15 ± 0.06 nmol min^−1^mg^−1^, expression of *cbrA* increased the uptake rate about 2fold (0.27 ± 0.09 nmol min^−1^mg^−1^). Next, we tested whether the sensor kinase domain is required for the stimulation of ^3^H-L-histidine transport by CbrA. We found that the SLC5 domain alone (amino acids 3 to 544 of CbrA) is sufficient to stimulate transport to about the same extent as the full-length protein. In addition, alteration of the predicted phosphorylation site (His766) in the DHp domain (CbrA-H766N) did not significantly impact the stimulatory effect of CbrA on ^3^H-L-histidine uptake (Fig. 2a).

To obtain more detailed information on the kinetics of ^3^H-L-histidine uptake catalyzed by CbrA and the SLC5 domain, initial rates of transport were determined at substrate concentrations ranging from 0.1 to 25 µM. Transport rates were corrected for background activity (rate without CbrA or SLC5 domain at a given substrate concentration). The resulting transport rates both for CbrA and for the SLC5 domain saturated with increasing substrate concentration, as expected for a transporter-dependent process (Fig. 2b). Data analysis according to Michaelis and Menten yielded apparent *K*_m_ and *V*_*max*_ values of 0.7 ± 0.2 µM and 0.27 ± 0.02 nmol mg^−1^min^−1^ (CbrA), and 0.58 ± 0.18 µM and 0.24 ± 0.02 nmol mg^−1^min^−1^ (SLC5 domain).

Energetic requirements of the CbrA-dependent transport were analyzed by measuring ^3^H-L-histidine uptake into *P. putida* LW1 in the presence of different ionophores and ions (Fig. 2c). Only the proton ionophores carbonyl cyanide *m*-chlorophenyl hydrazine (CCCP) and 2,4-dinitrophenol (DNP) led to an inhibition of the CbrA-dependent transport process. Other ionophores with specificity for potassium and/or sodium (valinomycin, nigericin, nonactin) had no significant impact on transport. Since the SLC5 domain is characteristic for members of the solute/sodium symporter family, we expected sodium to stimulate transport. However, comparison of the transport rates in sodium-free Tris/MES buffer with and without NaCl or KCl did not reveal any significant difference (Fig. 2a,c). Taken together, the results suggest that transport catalyzed by CbrA is an energy-dependent process. While it seems to depend on an (electro)chemical proton gradient, there is no evidence that an (electro)chemical sodium gradient can drive transport.

The substrate specificity of CbrA was tested by recording ^3^H-L-histidine uptake into *P. putida* LW1 transformed with plasmid pUCP-Tc-*cbrA* in the presence of a 100fold molar excess of potential substrates (Fig. 2d). All proteinogenic amino acids were tested, and only L-histidine led to a significant inhibition of the uptake of the ^3^H-labeled substrate. In addition, L-ornithine, imidazole, 3-amino-1,2,4-triazole, 1,2,4-triazolyl-3-alanine, and urocanate did not have significant effects on ^3^H-L-histidine uptake. Also acetate, that is recognized by CrbS^13^, another SLC5-containing sensorkinase of *Pseudomonas* species, had no influence on the transport process. The results suggest that CbrA-catalyzed transport is specific for L-histidine, with the imidazole ring and the carboxyl and amino groups being decisive for binding.

### The domains SLC5 and PAS of CbrA bind L-histidine

**Figure 3:**
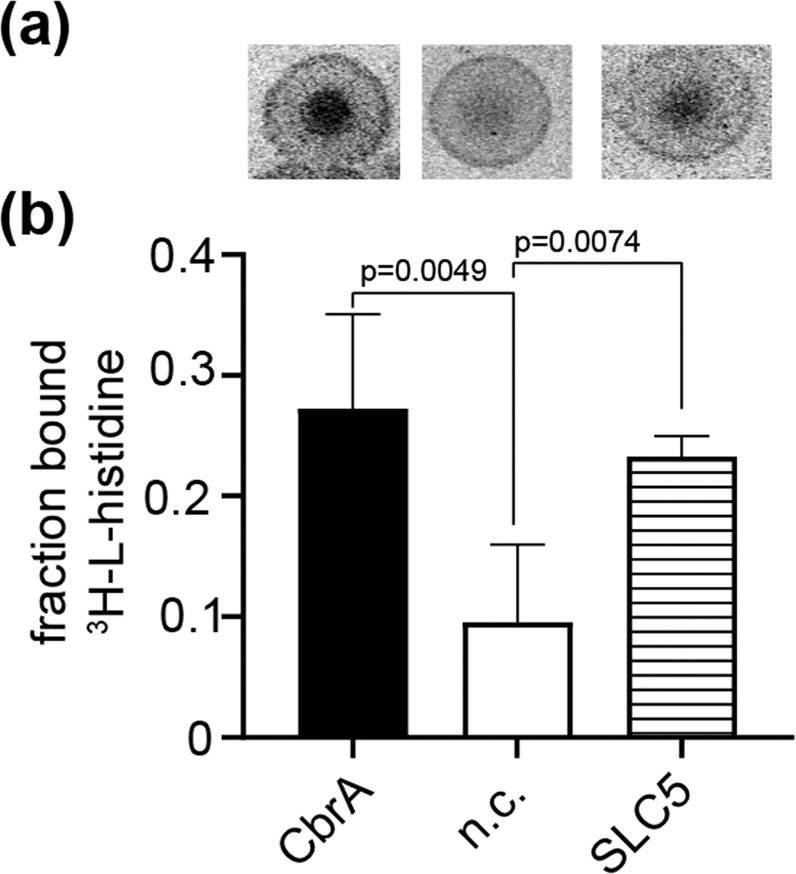
Binding of L-histidine to CbrA and the SLC5 domain of CbrA. Membrane vesicles were prepared from *E. coli* C43 containing either full length CbrA, the SLC5 domain, or none of the two proteins (negative control, n.c.). (**a**) The membrane vesicles were mixed with 1.35 µM ^3^H-L-histidine (37 Ci mmol^−1^) and spotted on nitrocellulose membranes. Diffusion of radioactivity in the resulting spots was visualized using a tritium screen and a Typhoon scanner. (**b**) The spots were quantified using ImageJ and the bound fraction of ^3^H-L-histidine was calculated according to Roelofs *et al*.^28^. The error bars represent standard deviation of four experiments. Welch’s t-test was applied for statistical analysis.

Based on the ability of CbrA to take up ^3^H-L-histidine, we set out to test binding of the amino acid to full-length CbrA, the SLC5 domain and the PAS domain (amino acids 614 to 745 of CbrA). Binding of ^3^H-L-histidine to CbrA and SLC5 domain in membrane vesicles was assessed using the DRaCALA assay^28^. For this purpose, genes encoding the respective proteins were cloned into pET21a, heterologously expressed in *E. coli* C43, and vesicles were prepared. Membranes isolated from *E. coli* C43 transformed with the empty plasmid pET21a served as negative control. The membrane vesicles were mixed with 1.35 µM ^3^H-L-histidine (37 Ci mmol^−1^) and spotted on nitrocellulose membranes. Diffusion of radioactivity in the resulting spots was visualized using a tritium screen and a Typhoon scanner (Fig. 3a). The observed retention of radioactivity in the center of the spot relative to the negative control was taken as evidence for binding of ^3^H-L-histidine to CbrA and the SLC5 domain (Fig. 3b). When cold L-histidine was added in excess, binding was reduced to the values observed for the negative control (not shown).

**Figure 4:**
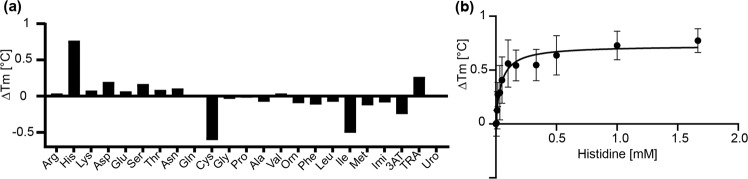
Binding of L-histidine to the PAS domain of CbrA. (**a**) The PAS domain was purified from *E. coli* C43 by Ni-NTA affinity chromatography, and the melting temperature *T*_*m*_ of the protein (170 µg ml^−1^ in 100 mM Tris-HCl pH7.5, 100 mM KCl and 10% glycerol) was determined in the presence of 1 mM of given amino acids and related compounds using NanoDSF. (**b**) The melting temperature *T*_*m*_ of the PAS domain was determined at given concentrations of L-histidine as described in (**a**). The error bars represent standard deviations of three experiments. All Δ*T*_*m*_ values are minimum 3fold larger than the respective standard deviations. Plotting of Δ*T*_*m*_ against the L-histidine concentration yielded a *k*_*d*_ value for L-histidine of 43 ± 13 µM.

To analyze ligand binding to the soluble PAS domain, the respective nucleotide sequence (plus six codons at the 3′ end encoding a 12His tag) was cloned into pET21a and expressed in *E. coli* BL21. The protein was purified by Ni-NTA affinity chromatography (Fig. S1a). Ligand binding was analyzed using thermal shift assays. Thereby, the impact of potential ligands on the melting temperature (*T*_*m*_) of the PAS domain was determined with Nano differential scanning fluorimetry (NanoDSF) in a Prometheus (NanoTemper). The method revealed an increase of the *T*_*m*_ value by 0.73 ± 0.13 °C, when 1 mM L-histidine was added to the protein solution (Fig. S2). The addition of 1 mM of other amino acids, imidazole, urocanate, 3AT or TRA did not affect the *T*_*m*_ value (Fig. 4a). Next, the effect of L-histidine on the *T*_*m*_ value was titrated by adding the amino acid at concentrations between 5 and 1667 µM. Plotting of Δ*T*_*m*_ against the L-histidine concentration led to a saturation curve and yielded a *k*_*d*_ value for L-histidine of 43 ± 13 µM (Fig. 4b). These results were verified using a different method to detect the *T*_*m*_, by adding SYPRO Orange that binds to hydrophobic regions of the protein (Fig. S3a). The *k*_*d*_ value for L-histidine determined with this method was 46 ± 17 µM (Fig. S3b).

Taken together, the results suggest that the membrane-integral SLC5 domain as well as the cytosolic PAS domain of CbrA can specifically bind L-histidine.

### Autophosphorylation of CbrA and phosphotransfer to CbrB

**Figure 5:**
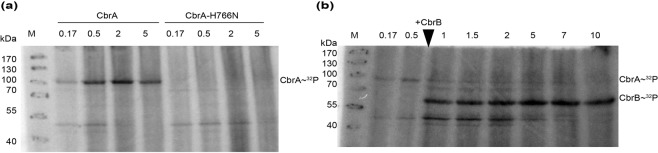
Phosphorylation of CbrA and phosphotransfer onto CbrB. (**a**) *E. coli* TKR2000 membrane vesicles containing either CbrA or CbrA-H766N (in 50 mM Tris-HCl, pH7.5, 10% glycerol, 10 mM MgCl_2_, 2 mM dithiothreitol, 360 mM KCl) were incubated with γ-^32^P-ATP. The reaction was stopped at given time points (min), and proteins were separated by SDS-PAGE. Radioactive protein bands were visualized using a phosphor screen. (**b**) Transfer of the phosphoryl group onto purified CbrB that was added after 45 sec of incubation of CbrA in membrane vesicles with γ-^32^P-ATP. CbrA has a predicted size of 109 kDa and CbrB of 54 kDa. Representative gels of three replicates are shown. Complete gels are presented in Fig. S4a,b.

To measure the putative autokinase activity of CbrA, the respective gene was heterologously expressed in *E. coli* TKR2000 (inactive F_o_F_1_ ATPase)^29^, and membrane vesicles were prepared. Vesicles with CbrA-H766N (putative site of phosphorylation was altered) served as negative control. The vesicles were incubated with γ-^32^P-ATP as a phosphate donor, then subjected to SDS-PAGE, and radioactivity was detected using a phosphor screen. Autophosphorylation was observed in 50 mM Tris-HCl, pH7.5 supplemented with 10% glycerol, 10 mM MgCl_2_, 2 mM dithiothreitol, and 360 mM KCl for wild type CbrA but not for CbrA-H766N (Fig. 5a). Maximum autophosphorylation was achieved within 30 s of incubation. A high concentration of potassium ions (*e.g*., 360 mM KCl) was required for the autokinase activity probably to simulate ionic conditions as present in the bacterial cytosol. On the contrary, NaCl did not stimulate autophosphorylation of CbrA.

Next, we tested the capability of CbrA to transfer the phosphoryl group to the response regulator CbrB. For this purpose, *cbrB* (plus six codons at the 3′ end encoding a 6His tag) was cloned into pET21a, and expressed in *E. coli* BL21. The protein was purified by Ni-NTA affinity chromatography (Fig. S1b). Purified CbrB was added to the autokinase assay described in the previous paragraph 45 s after its initiation. The experiment revealed that the phosphoryl group was rapidly transferred (within < 15 s) from CbrA to CbrB (Fig. 5b).

Since CbrA was shown to bind L-histidine, we analyzed the impact of the amino acid on the CbrA phosphorylation activities. L-histidine did neither affect the autokinase nor the phosphotransfer activities of CbrA (Fig. S4c,d). The lack of an effect of L-histidine leaves open the possibility that a yet to be identified intracellular metabolite is perceived by CbrA as a signal.

**Figure 6:**
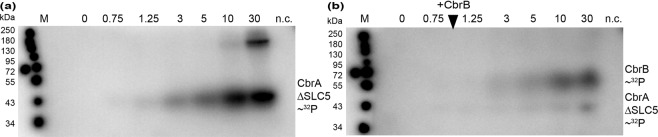
Phosphorylation of CbrA∆SLC5 and phosphotransfer onto CbrB. (**a**) Purified CbrA∆SLC5 (in 50 mM Tris-HCl, pH7.5, 10% glycerol, 10 mM MgCl_2_, 2 mM dithiothreitol, 360 mM KCl) was incubated with γ-^32^P-ATP. The reaction was stopped at given time points (min), and the protein was separated by SDS-PAGE. Radioactive protein bands were visualized using a phosphor screen. (**b**) Transfer of the phosphoryl group from CbrAΔSLC5 to purified CbrB that was added after 45 sec of incubation with γ-^32^P-ATP. Purified CbrAΔSLC5-H766N incubated with CbrB under the same conditions for 10 min served as negative control (n.c.). CbrAΔSLC5 has a predicted size of 44 kDa and CbrB of 54 kDa. Representative gels of three replicates are shown. Complete gels are presented in Fig. S5.

Is the membrane integral transporter domain SLC5 required for the phosphorylation activities of CbrA? To answer the question, the nucleotide sequence encoding only the cytoplasmic domains of CbrA (CbrAΔSLC5, amino acids 614 to 992 of CbrA plus twelve codons at the 3′ end encoding a 12His tag) was cloned into pET21a and expressed in *E. coli* C41. The soluble protein was purified by Ni-NTA affinity chromatography (Fig. S1c). Purified CbrAΔSLC5 (in 50 mM Tris-HCl, pH7.5 supplemented with 10% glycerol, 10 mM MgCl_2_, 2 mM dithiothreitol, and 360 mM KCl) catalyzed both autophosphorylation and transfer of the phosphoryl group to CbrB indicating that the SLC5 domain is not essential for these activities (Fig. 6a,b). However, while maximum autophosphorylation of wild type CbrA in membrane vesicles occurred within 30 s, soluble CbrAΔSLC5 needed about 10 min to reach the maximum value (Fig. 6a). Maximum phosphorylation of CbrB by CbrAΔSLC5 was reached within about the same period of time (about 10 min) and was probably limited by the low autokinase activity (Fig. 6b). Although a precise quantitative comparison of the activities of wild type CbrA in *E. coli* TKR2000 membrane vesicles and purified soluble CbrAΔSLC5 is not possible because the exact amount of wild type CbrA in the membranes is not known, the results seem to suggest that the SLC5 domain can modulate the autokinase activity of CbrA. Unfortunately, all trials to substitute the membrane vesicles by defined amounts of purified wild type CbrA in detergent or reconstituted into proteoliposomes failed due to the inactivity of the isolated protein under all test conditions. The results suggest that the SLC5 domain is important but not essential for the phosphorylation activities of CbrA.

### Phosphatase activity of CbrA

Besides phosphotransfer from sensor kinases, phosphorylation levels of response regulators can be modulated by autophosphorylation by small-molecule phosphodonors such as acetyl phosphate (ACP) and dephosphorylation by sensor kinases^30–32^. To test autophosphorylation of CbrB by ACP and a possible phosphatase activity of CbrA, we synthesized ^32^P-ACP from acetic anhydride and ^32^P-orthophosphate. ^32^P-ACP was then incubated with purified wild type CbrB and CbrB-D52N (Asp52 is the predicted site of phosphorylation) and the time course of phosphorylation was recorded for up to 90 min (Fig. S6). While ^32^P labeling of wild type CbrB was visible within 10 min of incubation, phosphorylation of CbrB-D52N was not observed within 90 min indicating that Asp52 is indeed the site of phosphorylation. Before testing a possible phosphatase activity of CbrA, excess of ^32^P-ACP was removed from the CbrB∼^32^P solution via a HiTrap desalting column. The resulting CbrB∼^32^P was incubated without and with CbrA in membrane vesicles prepared from *E. coli* TKR2000 or purified CbrAΔSLC5. Typical time courses of the dephosphorylation experiment are shown in Fig. S7. The percentage of radioactivity remaining at CbrB after 10 min of incubation relative to the zero-time point was used as a quantitative measure of dephosphorylation (Fig. S7). CbrB∼^32^P without additions was stable for minimum 10 min. Addition of CbrA containing membrane vesicles or of purified CbrAΔSLC5 did not significantly stimulate dephosphorylation of CbrB∼^32^P, and also the addition of L-histidine had no significant effect (Fig. S7b,e). In conclusion, CbrA did not show a significant phosphatase activity under the conditions of the experiments.

### Significance of the individual domains of CbrA for signal transduction

**Figure 7:**
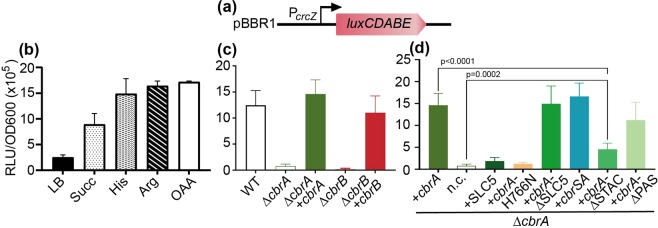
Analysis of CbrA/CbrB-dependent signal transduction using a P_*crcZ*_::*luxCDABE* reporter gene fusion. (**a**) Scheme of the reporter gene fusion P_*crcZ*_::*luxCDABE* plasmid pBBR1. (**b**) *P. putida* KT2440 Δ*cbrA* was co-transformed with plasmids pBBR1-P_*crcZ*_::*luxCDABE* and pUCP-Tc-*cbrA*. Cells were grown in LB medium (LB) or M9 medium supplemented with succinate (Suc), L-histidine (His), L-arginine (Arg), or oxaloacetate (OAA) as carbon sources under aerobic conditions at 30 °C. Luminescence (RLU) and optical density (OD_600_) were recorded over time in a microtiter plate reader. The RLU/OD_600_ ratios were taken as a measure of *crcZ* expression, and were calculated from values at the beginning of the exponential growth phase. (**c**) Expression of *crcZ* in *P. putida* KT2440 (wild type) and in *cbrA* and *cbrB* mutants. Cells were co-transformed with either pUCP-Tc, pUCP-Tc-*cbrA*, or pUCP-Tc-*cbrB*, and pBBR1-P_*crcZ*_::*luxCDABE*, grown in M9 medium with L-histidine as C-source, and RLU/OD_600_ ratios were determined as described in (**a**). (**d**) Expression of *crcZ* in *P. putida* KT2440 (Δ*cbrA*) transformed with pBBR1-P_*crcZ*_::*luxCDABE* and pUCP-Tc harboring the genetic information for wild type CbrA, the SLC5 domain of CbrA, CbrA-H766N, CbrAΔSLC5, the sensor kinase hybrid CbrSA, CbrAΔSTAC, or CbrAΔPAS. Mean values and standard errors were calculated from five independent experiments. All values were significantly different from the n.c. except for SLC5 and CbrA-H766N (p ≤ 0.0005).

To test signal transduction via CbrA/CbrB *in vivo*, a transcriptional reporter gene fusion was generated by fusing the promoter of one of the target genes (*crcZ*^14,15,23^) to the *luxCDABE* operon in plasmid pBBR1-MCS5*-lux*^33^. Genes *cbrA* (and its variants with given deletions) and *cbrB* were cloned into plasmid pUCP-Tc. Furthermore, genes *cbrA* and *cbrB* were individually deleted from the genome of *P. putida* KT2440 by homologous recombination using the suicide vector pMRS101^34^. The resulting mutants were co-transformed with plasmids pBBR1-P_*crcZ*_*::luxCDABE* and pUCP-Tc containing given *cbrA* or *cbrB* variants (Fig. 7a). To test the functionality of the reporter system, cells were grown on different carbon sources, and cell luminescence was determined. Expression of *crcZ* was (partially) repressed when cells were grown in LB medium or M9 minimal containing succinate, a preferred carbon source of *P. putida* (Fig. 7b). Less favorable carbon sources (L-histidine, L-arginine, oxaloacetate) led to maximum expression of *crcZ* as expected for a small RNA sequestering the Crc protein^15^ (Fig. 7b). Mutants with a deletion of either *cbrA* or *cbrB* did express *crcZ* only when transformed with pUCP-Tc-*cbrA* or pUCP-Tc-*cbrB* respectively, but not when pUCP-Tc was used (Fig. 7c). These results confirmed the functionality of the reporter system.

Next, we tested the impact of the deletion of individual domains of CbrA on *crcZ* expression with L-histidine as a carbon source (Fig. 7d). Contrary to wild type CbrA, CbrA-H766N did not activate expression of *crcZ* indicating that the conserved histidine in the DHp domain (site of phosphorylation) is essential for signal transduction. Consequently, also the membrane integral domain SLC5 alone did not induce *crcZ* expression. On the contrary, when the SLC5 domain of CbrA was deleted (CbrAΔSLC5) or replaced by the SLC5 domain of the homologous sensor kinase CrbS of *P. putida* KT2440 (CbrSA), *crcZ* expression was activated as observed with wild type CbrA. Deletion of the STAC domain (CbrAΔSTAC) allowed *crcZ* expression but at significantly reduced levels compared to wild type CbrA, while deletion of the PAS domain (CbrAΔPAS) had relatively little impact on *crcZ* expression (Fig. 7d).

## Discussion

The TCS CbrA/CbrB is known to participate in the regulation of the cellular carbon/nitrogen balance and to play a central role in carbon catabolite repression of *Pseudomonas* species^22–24,35^. Here, we analyze functional properties of the sensor kinase CbrA and its domains at the molecular level using the system of the soil bacterium *P. putida* KT2440 as a model. In agreement with a previous publication on CbrA of *P. fluorescence*^26^, we demonstrate that CbrA of *P. putida* KT2440 can catalyze the uptake of L-histidine. The apparent *K*_m_ of CbrA for L-histidine is with 0.7 µM similar to the *K*_m_ of PutP, another SLC5 family member, for L-proline^36^. The CbrA-dependent maximum rate of L-histidine uptake into cells (0.27 nmol mg^−1^ min^−1^) is relatively low but in the same range as the ones detected for other L-histidine transport systems of *Pseudomonas* species^26,37^. In fact, it was previously shown that CbrA supports growth of *P. fluorescence* on L-histidine when all other L-histidine transport systems are deleted^26^. Differing from PutP and other members of the SLC5 family, substrate uptake is not stimulated by sodium ions. This finding agrees with the observation that amino acids known to be involved in sodium binding (*e.g*., Ser340, Thr341 in the middle of transmembrane domain IX of PutP^36^) are not conserved in CbrA. Instead, studies with ionophores suggest that uptake is driven by an electrochemical proton gradient (Fig. 2b). In this case, CbrA would be the first functionally characterized member of the SLC5 family, whose transport activity is not stimulated by an electrochemical sodium gradient. Transport via the SLC5 domain is not affected by alterations in the sensor kinase domains of CbrA (*e.g*., CbrA-H766N) or by the complete removal of these domains. Similarly, other transporters associated with signal transductions systems (*e.g*., LysP, DctA, UhpC) were shown to catalyze transport independent of the interaction partner^2,38,39^.

Furthermore, our results suggest that besides the SLC5 domain also the PAS domain of CbrA can bind L-histidine. Both domains seem to be highly specific for L-histidine, and neither other amino acids nor structurally related molecules or degradation products of L-histidine are recognized (Figs. 2d and 4a). This result fits in principle with the concept of a dual-sensing receptor, which is able to detect and respond to both the availability of a substrate in the environment and the intracellular demand for this substrate^40^. However, since the CbrA/CbrB systems regulates the catabolism not only of L-histidine but of many different carbon and nitrogen sources (*e.g*., L-proline, L-arginine, xylose, mannose)^18^, the strict specificity for L-histidine is hard to understand. Instead, one would rather expect that a central metabolite acts as intracellular signaling molecule. If so, this metabolite has yet to be identified.

We show in *in vitro* experiments that CbrA autophosphorylates at the position of His766, and that the phosphoryl group is transferred to the response regulator CbrB. A CbrA-dependent dephosphorylation of CbrB~P is not observed. Despite the described binding of L-histidine to CbrA, the amino acid does not influence any of the three activities under our *in vitro* conditions. This finding further supports the idea that not L-histidine but a yet untested metabolite regulates the activities of CbrA. However, we cannot exclude that our *in vitro* test conditions do not allow detection of a L-histidine effect that might be relevant for the conditions in *P. putida* KT2440 cells.

The SLC5 domain of CbrA of *P. putida* KT2440 is not required for signal transduction. Neither substitution by the SLC5 domain of the homologous sensor kinase CrbS (regulates acetate utilization^13,40^) (CbrSA) nor complete removal of the SLC5 domain (CbrAΔSLC5) have a significant effect on the expression of the CbrA/CbrB target gene *crcZ* (Fig. 7d). Similar results were previously obtained with a CbrSA hybrid of *P. fluorescence*^13^. All the results agree with our finding that CbrAΔSLC5 has an autokinase activity and is capable of transferring the phosphoryl group to CbrB (Fig. 6). Nevertheless, a comparison of the time courses of autophosphorylation catalyzed by wild type CbrA and CbrAΔSLC5 seems to suggest that the autokinase activity is lower for the latter CbrA variant compared to wild type (Figs. 5 and 6). A reduced (deregulated) autokinase activity may explain the previously observed inhibition of growth on L-histidine of a *P. fluorescence* mutant expressing a *cbrAΔSLC5* variant^26^. Another publication reports that deletion of the “transmembrane domains” of CbrA reduces the expression of the CbrA/CbrB target gene PP_2810, and that the phenotype is reversed by overexpression of the soluble histidine kinase domain^14^. Taken together, all these observations suggest that although physical interactions between the SLC5 domain and the histidine kinase domain are not essential for signal transduction by CbrA, the SLC5 domain modulates the autokinase kinetics of the CbrA/CbrB system.

Furthermore, deletion of the STAC domain has a significant impact on signal transduction, albeit the domain is not essential for the activation of gene expression by the CbrA/CbrB system (Fig. 7d). Since it is assumed that the STAC domain mediates interactions between the SLC5 domain and the sensor kinase domain, the result further supports the idea that the SLC5 domain can modulate the phosphorylation kinetics of CbrA. Surprisingly, deletion of the PAS does not have a significant effect in signal transduction (Fig. 7d). A previous analysis revealed an impact of the PAS domain on CbrA-dependent gene expression^14^. The discrepancy may be explained by the different sizes of the deleted fragments and resulting effects on the remaining protein structure and functionality.

**Figure 8:**
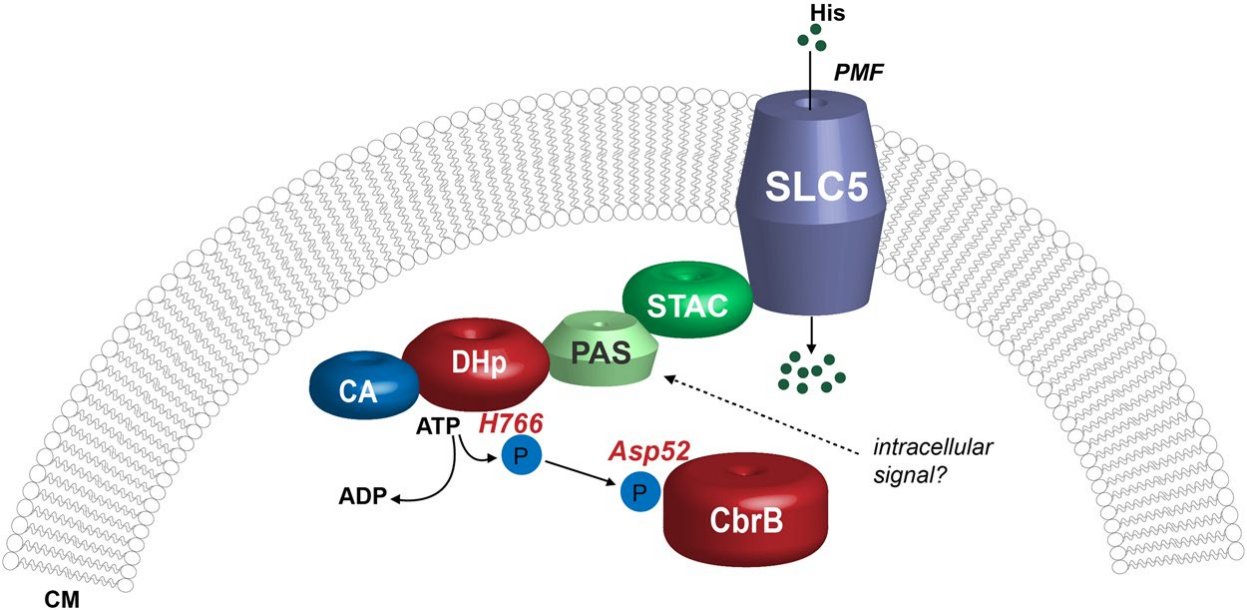
Proposed model of CbrA function. The SLC5 domain takes up L-histidine into the cell using an electrochemical proton gradient as driving force. The DHp domain autophosphorylates under ATP consumption at His766, and transfers the phosphoryl group to Asp52 of CbrB. The SLC5 domain may influence the autokinase and phosphotransfer activities. An impact of L-histidine on these activities is not observed. Instead, a yet to be identified intracellular metabolite may be perceived as a signal by the PAS domain. CM, cytoplasmic membrane; PMF, proton motive force (electrochemical proton gradient).

Taken together, we demonstrate here that CbrA of *P. putida* KT2440 can specifically bind and transport L-histidine using an electrochemical proton gradient as a driving force (Fig. 8). The significance of L-histidine for signal transduction remains enigmatic. First experimental evidence is presented suggesting that the transporter domain SLC5 via the STAC domain modulates the kinetics of autophosphorylation catalyzed by CbrA.

## Materials and Methods

### Strains and cultivation conditions

All strains of *P. putida* and *E. coli* used in this investigation are listed in Table S1. Cells were cultivated aerobically at 30 °C and 37 °C, respectively. When cells were transformed with plasmids, the respective antibiotics were added at the following concentrations: ampicillin/carbenicillin (100 µg ml^−1^), tetracycline (50 µg ml^−1^), gentamycin (30 µg ml^−1^), and kanamycin 50 (µg ml^−1^). For standard cultivation and precultures, LB medium was used (1% tryptone/peptone, 1% NaCl, 0.5% yeast extract). For plates, 1.5% agar was added to the medium and poured into petri dishes. For minimal medium, a M9 salt solution supplemented with 18.7 mM NH_4_Cl, 0.2 mM CaCl_2_, 2 mM MgSO_4_, 2 mM thiamine and 20 mM of the appropriate C-source were used. Additionally, the following trace elements were added: 134 µM Na_2_-EDTA, 31 µM FeCl_3_, 6.2 µM ZnCl_2_, 0.76 µM CuCl_2_, 0.42 µM CoCl_2_, 1.62 µM H_3_Bo_3_, 0.081 µM MnCl_2._

### Generation of strains and plasmids

To individually delete *cbrA* (PP_4695) and *cbrB* (PP_4696) from the genome of *P. putida* KT2440, the nucleotide sequences flanking these genes (500 base pairs) were cloned up- and downstream of a FRT-kanamycin resistance cassette in the suicide vector pMRS101^34^ followed by homologous recombination. Subsequently, the kanamycin resistance cassette was removed from the genome of the resulting mutants with FLP recombinase. *P. putida* KT2440 mutant LW1 (Δ*cbrA* Δ*hutTH* Δ*hutWX*) was created by cloning the flanking regions of the respective genes into the suicide vector pNPTS138-R6KT followed by double homologous recombination for insertion into the bacterial chromosomal genome^27^.

For complementation analyses, *cbrA* and its variants encoding CbrA without SLC5, STAC, PAS, or sensor kinase domain were amplified by PCR with primers listed in Table S3, digested with restriction enzymes *Nde*I and *Xho*I and cloned into pUCP-Tc. For overexpression, *cbrB*, *cbrA* or nucleotide sequences encoding individual domains of CbrA and a C-terminal His tag were cloned into pET21a with *Nde*I and *Xho*I. Primers used for amplification by PCR are listed in Table S3. For DNA extraction from agarose gels and purification of PCR products the HiYield® PCR Clean-up/Gel Extraction Kit (SLG®) was used. Plasmid extraction from 3 ml overnight cultures in LB was performed with the HiYield® Plasmid Mini Kit (SLG®). All plasmids used are listed in Table S2.

### Luminescence reporter assays

A *crcZ::luxCDABE* transcriptional reporter gene fusion was generated by PCR amplification of the promoter region of gene *crcZ* with primers PcrcZ_BamHI_s and PcrcZ_EcoRI_as (Table S3) and cloning of the resulting fragment into the *Bam*HI and *Eco*RI sites of plasmid pBBR1-MSC5*-lux*^33^. *P. putida* cells with a deletion of either *cbrA* or *cbrB* were co-transformed with plasmids pBBR1-*crcZ::lux* and pUCP-Tc containing given *cbrA* or *cbrB* variants. One hundred fifty µl LB or M9 minimal medium containing 30 µg ml^−1^ gentamycin, 50 µg ml^−1^ tetracycline and given carbon sources were pipetted per well of black 96well-plates (Corning) and inoculated with the respective *P. putida* strain from an overnight culture (start OD_600_ = 0.1, d = 1 cm). The plates were incubated shaking at 30 °C for 24–30 h in a CLARIOstar (BMG Labtech). OD_600_ and luminescence (RLU, relative light units) were measured every 30 min, and the RLU/OD_600_ ratios were determined.

### Whole cell transport measurement

*P. putida* LW1 containing pUCP-Tc plasmids with variants of *cbrA* were cultivated in LB medium as described and gene expression was induced by adding 0.5 mM IPTG at OD_600_ = 0.7 and continued incubation for 3 h. The cells were harvested and washed in Tris/MES buffer (pH6) and kept on ice. Two hundred µl aliquots of cell suspension with OD_600_ = 5.0 were energized with 10 mM D-lactate at 25 °C for 10 min. To initiate transport, ^3^H-L-histidine (500 Ci mol^−1^) was added to the cell suspension to a final concentration of 1 µM. After given periods of incubation at 25 °C, uptake was stopped by adding ice-cold stop buffer (0.1 M LiCl, 0.1 M KH_2_PO_4_, pH6.0) and rapid filtration through nitrocellulose filters (pore size 0.4 µm) with the aid of a vacuum pump. For competition analyses, given compounds (amino acids, L-histidine degradation products) were added simultaneously with ^3^H-L-histidine in 100fold molar access (final concentration 100 µM) to the cell suspension and ^3^H-L-histidine uptake was recorded as described. Ionophores were individually added to the cell suspension at the following final concentrations: 6 µM nigericin, 20 µM carbonyl cyanide *m*-chlorophenyl hydrazone (CCCP), 2 mM 2,4-dinitrophenol (DNP), 10 µM nonactin, 2 µM valinomycin. Radioactivity attached to the nitrocellulose filters was detected by liquid scintillation counting using a Tri-Carb 2910TR counter. As a standard, 1 µl of a 200 µM ^3^H-L-histidine solution (500 Ci mol^−1^) was applied. The total protein amount in the cell suspensions was determined by the Peterson Protein assay for whole cells^41^. Transport data were corrected for activity of cells without CbrA (negative control) and plotted according to Michaelis-Menten using GraphPad Prism.

### DRaCALA

CbrA or CbrA-SLC5 containing membrane vesicles were prepared from *E. coli* C43 heterologously expressing the respective genes from plasmid pET21a upon induction by 0.5 mM IPTG. Cells transformed with plasmid pET21a without *cbrA* served for the preparation of the negative control. Cells were disrupted with high pressure (1.35 kbar) in a Constant Cell Disruptor followed by ultracentrifugation at 235000 *g* and washing. Membrane vesicles were resuspended in 100 mM KP_i_ buffer pH7.5, and the amount of protein was determined by the Peterson protein assay^41^. For the differential radial capillary action of ligand assay (DRaCALA), the protocol of Roelofs *et al*.^28^ was followed. ^3^H-L-histidine (final concentration 1.35 µM, 37 Ci mmol^−1^) was added to the pre-incubated membrane vesicles containing 27 mg mL^−1^ total protein, and samples were incubated at 25 °C for 10 min. Five µL aliquots were subsequently pipetted onto dry nitrocellulose (GE Healthcare) in triplicates. The nitrocellulose was exposed to a Storage Tritium Screen, and a Typhoon Trio Imager (Amersham Biosciences) was used for detection of radioactivity. Analysis of the resulting image was performed with ImageJ.

### Protein purification

Genes encoding CbrA, CbrA∆SLC5, CbrA∆SLC5-H766N, CbrA-PAS, or CbrB were expressed from plasmid pET21a in *E. coli* BL21 or C43. For this purpose, an over day preculture was used to inoculate a 100 ml overnight culture, which in turn was used to inoculate a 1 l culture. Gene expression was induced by adding 0.5 mM IPTG at OD_420_ = 1. Cells were harvested, washed (0.1 M KPi pH7.5) and the pellets stored at −80 °C after freezing in liquid nitrogen. All following steps were carried out at 4 °C or on ice. The cells were resuspended in the respective purification buffer (0.2 g ml^−1^) and disrupted with high pressure (1.35 kbar) in a Constant Cell Disruptor. For purification of soluble proteins, the cell lysates were centrifuged first at low speed (4500 *g*) to remove cell debris and then at high speed to remove the membrane fraction (235000 *g*). The cytosolic fraction was applied to a HisTrap column on an Äkta system, thoroughly washed with a 10–50 mM imidazole gradient and eluted with 250 mM imidazole. Alternatively, the same steps were carried out manually on a chromatography column. The purity of the proteins was estimated via Coomassie-stained SDS-PAGE and the identity by Western Blot with a α-PentaHis Antibody. The protein concentration was measured by the NanoDrop or via Bradford protein assay^42^. The buffer for the cytosolic domain (CbrA∆SLC5) contained 100 mM Tris-HCl pH7.5, 100 mM KCl and 10% glycerol. The buffer for the PAS domain contained 100 mM Tris-HCl pH7.5, 100 mM KCl and 10% glycerol for NanoDSF assays or 100 mM KPi buffer pH7.5 and 20% glycerol for thermal shift assays with SYPRO Orange. The buffer for CbrB contained 50 mM Tris-HCl pH7.5, 100 mM NaCl and 5% glycerol.

To isolate wild type CbrA, cell lysates in 50 mM Tris-HCl pH7.5, 300 mM KCl, 10% glycerol were centrifuged first at low speed (4500 *g*) to remove cell debris and then at high speed to (235000 *g*) at 4 °C to obtain the membranes. The membrane pellet was washed, resuspended in a small volume of 50 mM Tris-HCl pH7.5, 300 mM KCl, 10% glycerol and if required stored in aliquots at −80 °C after shock freezing in liquid nitrogen. The protein amount in the membranes was determined via Peterson Protein assay^41^. The membrane proteins (5 mg ml^−1^ total membrane protein) were solubilized by adding 1.5% n-dodecyl β-D-maltoside during stirring for 30 min. The membranes were removed by ultracentrifugation (113000 *g*). The solubilized proteins were mixed with Ni-NTA resin for 45 minutes and packed onto a chromatography column. After washing with imidazole (10 and 40 mM) CbrA was eluted with 400 mM imidazole. Purity and concentration were estimated and measured as for soluble proteins. The buffer for CbrA contained 50 mM Tris-HCl pH7.5, 300 mM KCl, 10% glycerol and 0.04% n-dodecyl β-D-maltoside. Imidazole was removed from the purified proteins either via gel filtration or dialysis. Purified CbrA was reconstituted into liposomes prepared from Avanti *E. coli* polar lipid extract at a lipid to protein ratio (w/w) of 50 to 1. Reconstitution was carried out as previously described for *E. coli* PutP^43^.

### Determination of protein melting temperature

Two separate methods were used to measure the melting temperature (*T*_*m*_) of the purified PAS domain. One method was based on Nano differential scanning fluorimetry (NanoDSF)^44^ and used the Prometheus system from Nanotemper. The latter system recorded the intrinsic tryptophan and tyrosine fluorescence. The ratio of the fluorescence intensities at 350 nm and 330 nm was determined while the temperature was steadily increased from 20 to 95 °C which results in a melting curve. The inflection point of the melting curve is considered as the *T*_*m*_. Ligand binding was analyzed by determining the impact of potential ligands on the *T*_*m*_ value. As a second method, the fluorescent dye SYPRO orange was added to the protein and the fluorescent signal was measured in a real-time PCR instrument (Bio-Rad iCycler5) while the temperature was steadily increased from 10 to 80 °C. The dye binds preferentially to hydrophobic regions resulting in an increase in fluorescence emission while the protein unfolds and hydrophobic parts become exposed^45,46^. The ∆ *T*_*m*_ is calculated by comparing the *T*_*m*_ of the respective sample to a control without ligand.

### Autokinase and phosphotransfer activity assays

Nucleotide sequences encoding CbrA and its variants were heterologously expressed in *E. coli* TKR2000 (F_o_F_1_ ATPase inactivated)^29^ from pBAD24, and membrane vesicles were prepared and suspended in 50 mM Tris-HCl, pH7.5 supplemented with 2 mM DTT, 10 mM MgCl_2_, and 360 mM KCl to yield a final protein concentration of 150–200 µg ml^−1^. If indicated, L-histidine was added to a final concentration of 1 mM. Phosphorylation was initiated by adding 20 µM γ-^32^P-ATP (4760 Ci mol^−1^), 100 µM γ-^32^P-ATP (956 Ci mol^−1^) or 0.05 µM γ-^32^P-ATP (3640 Ci mmol^−1^) (Amersham, Bioscience). The samples were incubated at 30 °C and after given periods of time stopped by mixing with 5x SDS-loading dye solution. For the transfer onto the response regulator, purified CbrB (500 µg ml^−1^) was added after 45 s of the incubation of CbrA with γ-^32^P-ATP. All samples were loaded onto a 10% SDS gel and run at 100 V for 3 h. Gels were dried on Whatman paper, wrapped in sticky foil and exposed to a phosphor screen (GE Healthcare) overnight. The screen was scanned in a Typhoon scanner.

### Phosphatase activity assay

^32^P-ACP was synthesized from ^32^P-orthophosphate (Hartmann Analytic) with 2 mCi activity on the reference day (10 mCi ml^−1^). The synthesis was performed as described by Stadtmann (1957)^47^. The amount was measured using the assay by Lipmann and Tuttle^48^ and found to be approximately 140 µmol in total. The yield was calculated by measuring the CPM of the starting material and the product in a scintillation counter, which enabled us to estimate the specific radioactivity with approximately 12 Ci mol^−1^.

To phosphorylate CbrB, the purified protein was mixed with ^32^P-ACP in 50 mM Tris-HCl, pH7.5, 100 mM KCl, 10% glycerol, 20 mM MgCl_2_ and incubated at 30 °C. The phosphorylation reaction was terminated at a given time point (usually 60 minutes) by changing the buffer in a desalting column (HiTrap, GE Healthcare) equilibrated with 50 mM Tris-HCl, pH7.5, 360 mM KCl, 2 mM DTT, 10 mM MgCl_2_ to remove excess ^32^P-ACP. This dilutes the protein 2fold resulting in a final protein amount of ~0.4 mg ml^−1^.

To test the capability to dephosphorylate CbrB~P, CbrA in TKR200 membrane vesicles (3 mg ml^−1^) or purified CbrA∆SLC5 (0.38 mg ml^−1^) was added to the CbrB~P solution (0.4 mg ml^−1^) in 50 mM Tris-HCl, pH7.5, 360 mM KCl, 2 mM DTT, 10 mM MgCl_2_. If required 1 mM L-histidine was added to the buffer. The samples were incubated at 30 °C and the reaction terminated by adding 5x SDS loading dye solution. For the control, buffer was added instead of CbrA to test the stability of phosphorylation. The samples were further treated as for the kinase assay.

## Supplementary information

Supplementary Information.

## Data Availability

The data that support the findings of this study are available from the corresponding author upon reasonable request.
