# Supplementary material for: Transport and kinase activities of CbrA of Pseudomonas putida KT2440
Source: Sci Rep. 2020 Mar 25;10:5400. doi: 10.1038/s41598-020-62337-9 (PMC7096432; doi:10.1038/s41598-020-62337-9)
Supplement: Supplementary file 1 — Supplementary Information. [file 41598_2020_62337_MOESM1_ESM.pdf]

Supplementary Information

Transport and kinase activities of CbrA of *Pseudomonas putida* KT2440

Larissa Wirtz[1], Michelle Eder[1], Kerstin Schipper[1*], Stefanie Rohrer[1**] and Heinrich Jung[1‡]

[1]Division of Microbiology, Department of Biology 1, Ludwig Maximilians University Munich, D-82152 Martinsried, Germany

[‡]Correspondence and requests for materials can be addressed to Heinrich Jung, hjung@lmu.de

*Current address: Institute of Microbiology, Department of Biology, Heinrich-Heine-University, D-40225 Düsseldorf, Germany

** Current address: Technical University of Munich, D-80333 Munich, Germany

Materials included:

Table S1. Strains used in this investigation.

Table S2. Plasmids used in this investigation.

Table S3. Oligonucleotides used in this investigation.

Figure S1. SDS-PAGE analysis of the courses of purification of CbrA domains and CbrB

Figure S2. Determination of the melting temperature (T_m) of CbrA-PAS via nanoDSF.

Figure S3. Determination of the melting temperature (T_m) of CbrA-PAS via SYPRO Orange.

Figure S4. Phosphorylation of CbrA and phosphotransfer onto CbrB (complete gels)

Figure S5. Phosphorylation of CbrAΔSLC5 and phosphotransfer onto CbrB (complete gels)

Figure S6. Phosphorylation of CbrB and CbrB-D52N by acetyl phosphate.

Figure S7. Test of a possible CbrA-dependent dephosphorylation of CbrB~^{32}P.

Supplementary References

Table S1. Strains used in this investigation

**Strain**	**Description**	**Reference**
Escherichia coli BL21(DE3)	*E. coli* B *dcm ompT hsdS(rB-mB) gal*	Novagen
Escherichia coli BL21(DE3) pLysS	*E. coli* B *dcm ompT hsdS(rB-mB) gal pLysS* Cm^R	Novagen
Escherichia coli C41	*E. coli* B F– *ompT gal dcm hsdSB(rB- mB-)(DE3)*	Lucigen
Escherichia coli C43	*E. coli* F– *ompT gal dcm hsdSB(rB- mB-)(DE3)*	Lucigen
Escherichia coli TKR2000	*ΔkdpFABCDE thi rha lacZ nagA trkA405 trkD1 atp706*	[1,2]
Pseudomonas putida KT2440	*rmo- mod+*	[3,4] *DSMZ 6125, ATCC 47054*
Pseudomonas putida KT2440 *ΔcbrA*	*ΔcbrA*	This work
Pseudomonas putida KT2440 *ΔcbrB*	*ΔcbrB*	This work
Pseudomonas putida KT2440 LW1	*ΔcbrA ΔhutT ΔhutH Δ3558 Δ3559*	This work

Table S2. Plasmids used in this investigation

**Plasmid**	**Description**	**Reference**
pMRS101	*LMBP 3654,* suicide vector*; ApR, SmR, sacB*	[5]
pFLP2	*ApR; Flp recombinase, sacB*	[6]
pNPTS138-R6KT	*mobRP4*$^+$*ori*-R6K *sacB*; suicide plasmid for in-frame deletions; Km^r (npt1)	[7]
pUCP-*Nde*I	pUCP19 with *Nde*I restriction enzyme cloning site, Amp^R	[8]
pUCP-*Nde*I-Tc^R	*tet* in pUCP-*Nde*I via *SspI*, $Amp^{R,}$ Tc^R	This work
pUCP*cbrB*-6his-Tc^R	*cbrB,* in pUCP-*Nde*I-Tc^R, 6-his-tag, Amp^R, Tc^R	This work
pUCP*cbrA*-6his-Tc^R	*cbrA* (Δ1-6) in pUCP-*Nde*I-Tc^R, 6-his-tag, Amp^R, Tc^R	This work
pUCP*cbrA*-SLC5-6his-Tc^R	*cbrA*-SLC5 (Δ1-6,Δ1633-2976) in pUCP-Nde-Tc^R, 6His tag, Amp^R, Tc^R	This work
pUCP*cbrA*-ΔSLC5-6his-Tc^R	*cbrA*-ΔSLC5 (Δ1-1509) in pUCP-*Nde*I-Tc^R, 6His tag, Amp^R, Tc^R	This work

pUCP*cbrA*-H766N-6his-Tc^R	*cbrA*-H766N (Δ1-6, H766N) in pUCP-NdeI-Tc^R, 6His tag, Amp^R, Tc^R	This work
pUCP-SA-6his-Tc^R	*crbS* (1-1598*)* + *cbrA* (1539-2976) in pUCP-*Nde*I-Tc^R, 6His tag, Amp^R, Tc^R	This work
pUCP*cbrA*-ΔSTAC-6his-Tc^R	*cbrA*-ΔSLC5 (Δ1-6, Δ1588-1770) in pUCP-*Nde*I-Tc^R, 6His tag, Amp^R, Tc^R	This work
pUCP*cbrA*-ΔPAS-6his-Tc^R	*cbrA*-ΔSLC5 (Δ1-6, Δ1894-2211) in pUCP-*Nde*I-Tc^R, 6His tag, Amp^R, Tc^R	This work
pET21a	N-terminal T7-tag, C-terminal His-tag, Amp/Carb^R, lacI	Novagen
pE*cbrA*-2-12His	*cbrA*-2 (Δ1-6) in pET21a, C-terminal 12His tag	This work
pE*cbrA*-2-SLC5-12His	*cbrA*-2-SLC5 (Δ1-6, Δ1632-2976) in pET21a, C-terminal 12His tag	This work
pE*cbrA*-PAS-12His	*cbrA*-PAS (1840-2235) in pET21a, C-terminal 12His tag	This work
pE*cbrA*- ΔSLC5-12His	*cbrA*- ΔSLC5 (1840-2976) in pET21a, C-terminal 12His tag	This work
pE*cbrA*- ΔSLC5-12His-H766N	*cbrA*- ΔSLC5 (1840-2976, H766N) in pET21a, C-terminal 12His tag	This work
pE*cbrB*-6His	*cbrB* in pET21a, C-terminal 6-his-tag	This work
pE*cbrB*-D52N-6His	*cbrB* in pET21a (D52N), C-terminal 6His tag	This work
pBAD24	Amp^R, *araC* promoter	[9]
pB*cbrA*-12his	*cbrA* in pBAD24, 12His tag	This work
pBBR1-MSC5 *luxCDABE*	*luxCDABE* and terminators lambda T0 rrnB1 T1 cloned into pBBR1-MCS5 for plasmid-based transcriptional fusions; Gmr	[10,11]
pBBR1-*lux*-P$_{crcZ}$	*luxCDABE* under the control of the *crcZ* promoter	This work

Table S3. Oligonucleotides used in this investigation. Nucleotides marked in red are restriction sites.

**Oligonucleotide**	**Sequence (5'->3')**
Del4695A1s	TTACCTGCAGAATTCGGGCCCCGTGCAGCCGGA
Del4695A2as	CACCATGAGGGATCCGGCGCTGATCAG
Del4695B1s	CCGAAGACAGTCGACTCATCGAAAGCC
Del4695B2as	TGATCGGGTAAGCTTACTAGTGGGCCACGCCGG

delcbrBA1s	GCCTGCCGAATTCGGGCCCTGCGCGAGGAAC
delcbrBA2as	GACGATGGTACCGTGCGGCATTGA
delcbrBB1s	CGACTGCACGTCGACGCCCTGAAGCTG
delcbrBB2as	TACGGCGGAAAGCTTACTAGTCAGATTTTTACG
Del5031A_s	TTCCTGGTGGAATTCGGTGGGGTCAAC
del5032_Bas	TTCCAGGGCGAATTCGCACGTATCTGC
delhutTH_Aas	GCCTGGCACCCCGGTAGCGGAGCCGGCCGAGCTG
delHutTH_Bs	CCGCTACCGGGGTGCCAGGCTTGAGGGTGAGTTCGG
del35589_As_BamHI	GCGCACTCAGGATCCGGTGGAAGAAAC
del3558-9_Aas_OL	GCAACGAGGGGTCGACAAAATGGATAGGCG
del3558-9_Bs_OL	CGCCTATCCATTTTGTCGACCCCTCGTTGC
del35589_Bas_NheI	TCATAGGCAGCTAGCAGGCTTTCGGCG
PP4696 s	GTCGAGAGAGGATCCCATATGCCGCACATTCTG
PP4696 as	GGCGTGCGAAGCTTAGCTCGAGGCTTCGCTGGTAGCGTT
1935 as	CAGGCAACTATGGATGAACGA
SDcbrBs	GTCCCCGGATCCTCGAGACCGTCGAGAGAATTGAACATATGCC GCACATTC
4695-2 s	ATAGATATGCATATGAGCTTTAGC
4695_6800as	GCGCAGTGCTTGCTCGACTTC
4695_SSF3as	CAGTGCTTGCTCGAGTTCCTTTTGCGC
pE4695_XhoIchange_as	ATGATGATGGCTCGAGGCATTCTCTCGACG
SSF3 XhoI3	ATGATGGATGGCTCGAGACTTCCTTTTGCGC
CS-long_s_NdeI	GAAGAGGTCCATATGGCCGAAGCCTG
pp4695cs_XhoI_as	ATGATGATGCTCGAGATTCTCTCGACG
pp4695 cs ndeI_s	TCGAAAGCCATATGGAAGACTACCACT
pp4695_PAS_as	CTTGTCCTCGAGTGCCTGGGTTTC
4695_H766N_as	GCCGATCTCGTTGGCCACGCCGGC
4695_H766N_s	CGGCGTGGCCAACGAGATCGGCAAC

CrbS_OL_as2	CTCTCGGCGCTGTGGACGTGCACTGGTCTG
cbrA_OL_s2	CAGACCAGTGCACGTCCACAGCGCCGAGAG
PcrcZ_BamHI_s	AGCGAATAAGGATCCTACGCACCGCAC
PcrcZ_EcoRI_as	TGTACCAAGAATTCAGCAGGTGCCGTG

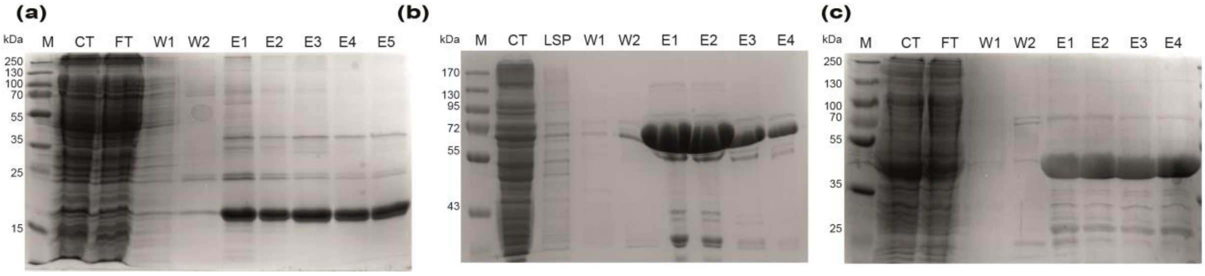

Figure S1. SDS-PAGE analysis of the courses of purification of CbrA domains and CbrB. Genes were expressed from plasmid pET21a in given *E. coli* strains. Cells were grown in three liters LB medium, disrupted by high pressure treatment, and His-tagged proteins were isolated by Ni-NTA affinity chromatography. Aliquots of the fractions were subjected to SDS-PAGE and stained with Coomassie. (**a**) The PAS domain of CbrA (17 kDa including C-terminal 12His tag) was purified from *E. coli* BL21 (DE3) cells via HisTrap. (**b**) CbrB (54 kDa including C-terminal 6His tag) was purified from *E. coli* BL21 (DE3) plysS via HisTrap. (**c**) The cytosolic domain of CbrA (44 kDa including C-terminal 12His tag) was purified from *E. coli* C41 cells via HisTrap. M, marker/protein ladder; CT, cytosolic fraction; FT, column flow through; LSP, low speed centrifugation pellet; W1, wash buffer 1 with 10 mM imidazole; W2, wash buffer 2 with 30-50 mM imidazole; E, elution fractions with 250 mM imidazole.

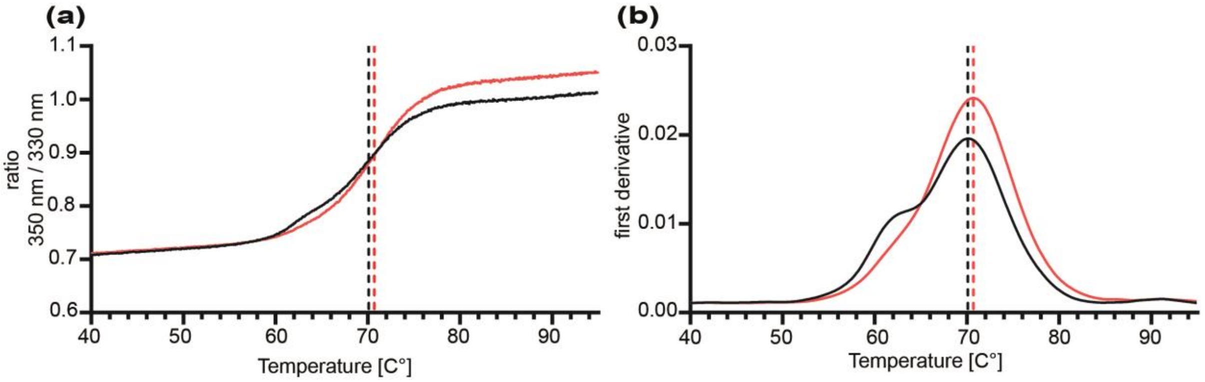

Figure S2. Determination of the melting temperature (T_m) of CbrA-PAS via Nano differential scanning fluorimetry in a Prometheus (Nanotemper). **(a)** The ratio 350 nm /330 nm results in a melting curve. **(b)** The peak of the first derivative shows the inflection point of the ratio curve (dotted line) which reflects the T_m. Exemplary melting curves of the PAS domain without ligand (black) and with 1.0 mM L-histidine (red) are shown (mean of a technical triplicate). The addition of L-histidine leads to a red shift of the fluorescence signal.

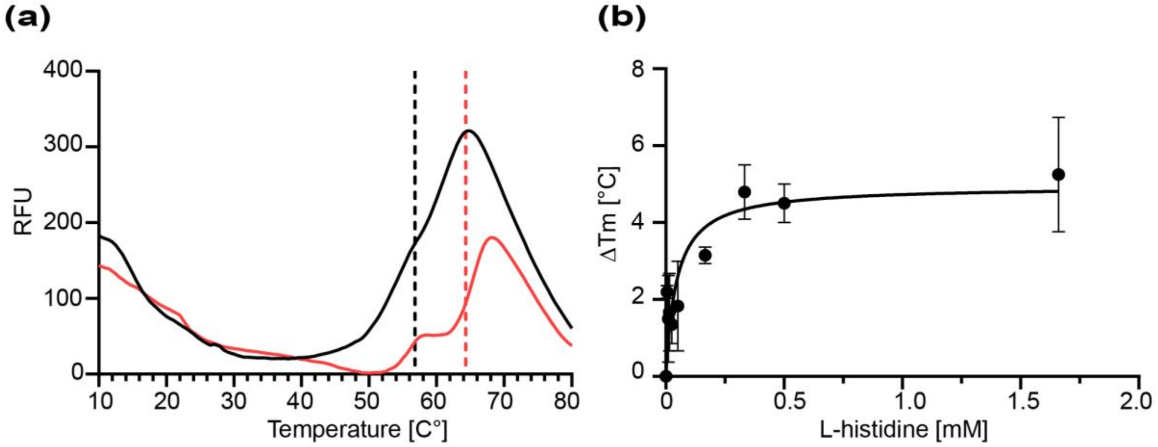

Figure S3. Determination of the melting temperature (T_m) of CbrA-PAS via SYPRO Orange. The relative fluorescent units (RFU) were recorded in a real-time PCR instrument while the temperature was steadily increased from 10 to 80°C. The dye binds to hydrophobic regions of the protein that are exposed while it unfolds. **(a)** Exemplary melting curves of the PAS domain without ligand (black) and with 1.66 mM L-histidine (red) are shown. The inflection point of the melting curve represents the T_m. **(b)** The ΔT_m was plotted against the ligand concentration and a Michaelis-Menten fit applied. The k_d value for L-histidine determined with this method was 46±17 µM.

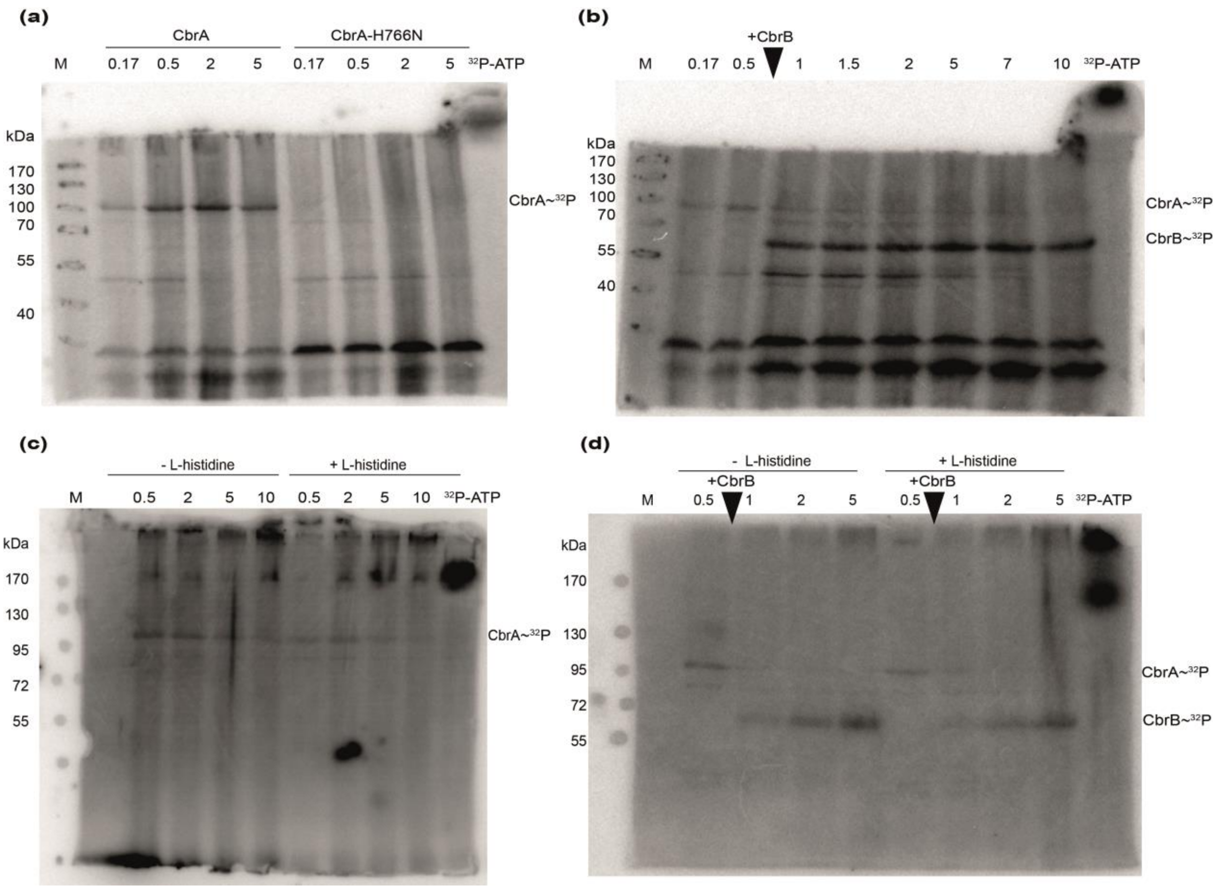

Figure S4. Phosphorylation of CbrA and phosphotransfer onto CbrB (complete gels accompanying Fig. 5). (**a**) *E. coli* TKR2000 membrane vesicles containing either CbrA or CbrA-H766N (in 50 mM Tris-HCl, pH7.5, 10% glycerol, 10 mM $MgCl_2$, 2 mM dithiothreitol, 360 mM KCl) were incubated with γ-^{32}P-ATP. The reaction was stopped at given time points (min), and proteins were separated by SDS-PAGE. Radioactive protein bands were visualized using a phosphor screen. (**b**) Transfer of the phosphoryl group onto purified CbrB that was added after 45 s of incubation of CbrA in membrane vesicles with γ-^{32}P-ATP. CbrA has a predicted size of 109 kDa and CbrB of 54 kDa. (**c**) *E. coli* TKR2000 membrane vesicles containing either CbrA (in 50 mM Tris-HCl, pH7.5, 10% glycerol, 10 mM $MgCl_2$, 2 mM dithiothreitol, 360 mM KCl) were incubated with γ-^{32}P-ATP either without or with the addition of 1 mM L-histidine. (**d**) Transfer of the phosphoryl group onto purified CbrB. CbrB was added after 45 s of incubation of CbrA in membrane vesicles with γ-^{32}P-ATP either without or with the addition of 1 mM L-histidine. ^{32}P-ATP, γ-^{32}P-ATP standard for quantification of radioactivity.

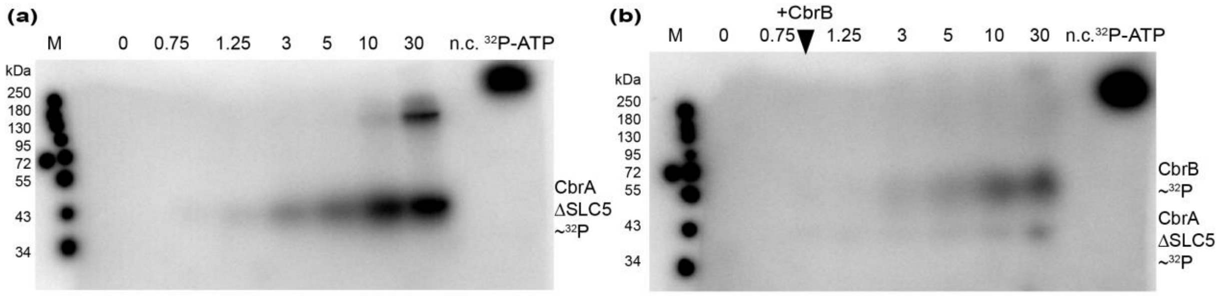

Figure S5. Phosphorylation of CbrAΔSLC5 and phosphotransfer onto CbrB (complete gels accompanying Fig. 6). (**a**) Purified CbrAΔSLC5 (in 50 mM Tris-HCl, pH7.5, 10% glycerol, 10 mM $MgCl_2$, 2 mM dithiothreitol, 360 mM KCl) was incubated with γ-^{32}P-ATP. The reaction was stopped after given periods of incubation (min), and the protein was separated by SDS-PAGE. Radioactive protein bands were visualized using a phosphor screen. (**b**) Transfer of the phosphoryl group from CbrAΔSLC5 to purified CbrB that was added after 45 s of incubation with γ-^{32}P-ATP. Purified CbrAΔSLC5-H766N incubated with CbrB under the same conditions for 10 min served as negative control (n.c.). CbrAΔSLC5 has a predicted size of 44 kDa and CbrB of 54 kDa. ^{32}P-ATP, γ-^{32}P-ATP standard for quantification of radioactivity.

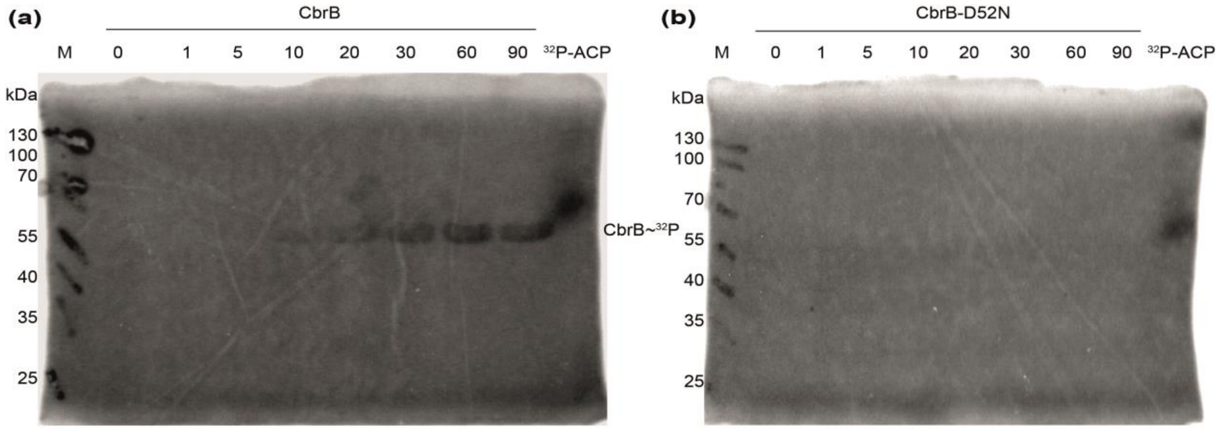

Figure S6. Phosphorylation of CbrB by acetylphosphate (ACP). (**a**) Purified CbrB and (**b**) CbrB-D52N (predicted site of phosphorylation replaced) were chemically phosphorylated with ^{32}P-ACP in 50 mM Tris-HCl, pH7.5, 100 mM KCl, 10% glycerol, 20 mM $MgCl_2$ and incubated at 30°C. Samples were taken after given periods of incubation (min) and applied to SDS-PAGE. Radioactive protein bands were visualized using a phosphor screen. ^{32}P-ACP, ^{32}P-ACP standard for quantification of radioactivity.

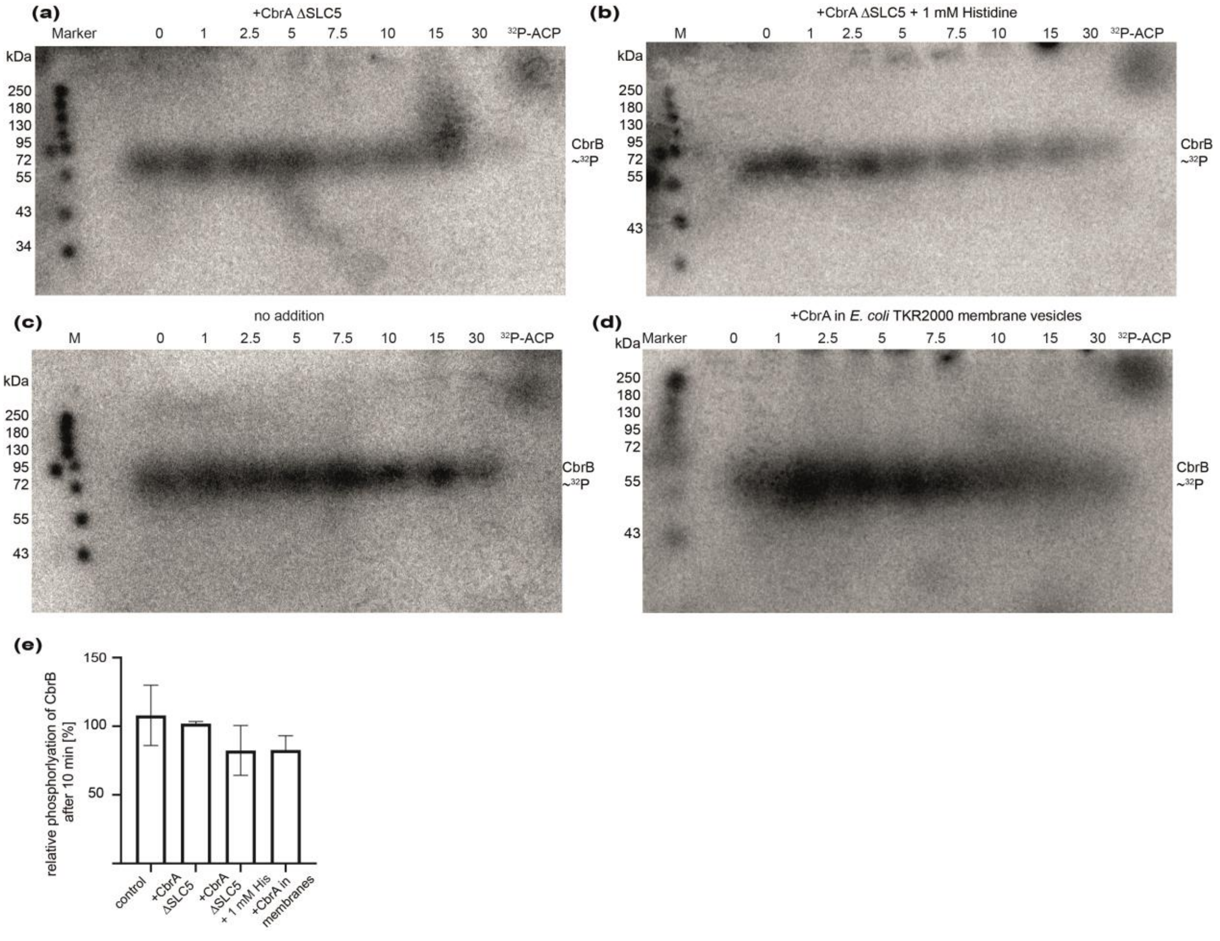

Figure S7. Test of a possible CbrA-dependent dephosphorylation of CbrB~^{32}P. Purified CbrB was chemically phosphorylated with ^{32}P-ACP as shown in Fig. S4. Given CbrA variants were added as indicated, and samples were taken after given periods of incubation (min) and applied to SDS-PAGE. Radioactive protein bands were visualized using a phosphor screen. (**a**) CbrB~^{32}P was incubated with purified CbrAΔSLC5 (in 50 mM Tris-HCl, pH7.5, 360 mM KCl, 2 mM DTT, 10 mM $MgCl_2$) (**b**) CbrB~^{32}P was incubated as in (a) with the addition of 1 mM L-histidine. (**c**) Purified CbrB~^{32}P was incubated with no CbrA added. (**d**) CbrB~^{32}P was incubated with full length CbrA contained in *E. coli* TKR2000 membranes. (**e**) The band intensity of the 10 min time point was measured and compared to the intensity at 0 min. Incubation of CbrB~^{32}P without a CbrA variant served as control (cp. also Fig. S5c). The relative quantification is based on three replicates. CbrB has a predicted size of 54 kDa. Complete gels of representative experiments are shown.; ^{32}P-ACP, ^{32}P-ACP standard for quantification of radioactivity.

Supplementary References

1 Kollmann, R. & Altendorf, K. ATP-driven potassium transport in right-side-out membrane vesicles via the Kdp system of *Escherichia coli*. *Biochim. Biophys. Acta* **1143**, 62-66, doi:https://doi.org/10.1016/0005-2728(93)90216-3 (1993).

2 Siebers, A. & Altendorf, K. The K+-translocating Kdp-ATPase from *Escherichia coli*. *Eur. J. Biochem.* **178**, 131-140, doi:10.1111/j.1432-1033.1988.tb14438.x (1988).

3 Regenhardt, D. *et al.* Pedigree and taxonomic credentials of *Pseudomonas putida* strain KT2440. *Environ. Microbiol.* **4**, 912-915, doi:10.1046/j.1462-2920.2002.00368.x (2002).

4 Bagdasarian, M. *et al.* Specific-purpose plasmid cloning vectors II. Broad host range, high copy number, RSF 1010-derived vectors, and a host-vector system for gene cloning in *Pseudomonas*. *Gene* **16**, 237-247, doi:http://dx.doi.org/10.1016/0378-1119(81)90080-9 (1981).

5 Sarker, M. R. & Cornelis, G. R. An improved version of suicide vector pKNG101 for gene replacement in Gram-negative bacteria. *Mol. Microbiol.* **23**, 410-411, doi:10.1046/j.1365-2958.1997.t01-1-00190.x (1997).

6 Hoang, T. T., Karkhoff-Schweizer, R. R., Kutchma, A. J. & Schweizer, H. P. A broad-host-range Flp-FRT recombination system for site-specific excision of chromosomally-located DNA sequences: application for isolation of unmarked *Pseudomonas aeruginosa* mutants. *Gene* **212**, 77-86, doi:https://doi.org/10.1016/S0378-1119(98)00130-9 (1998).

7 Lassak, J., Henche, A.-L., Binnenkade, L. & Thormann, K. M. ArcS, the cognate sensor kinase in an atypical Arc system of *Shewanella oneidensis* MR-1. *Appl. Environ. Microbiol.* **76**, 3263-3274, doi:10.1128/AEM.00512-10 (2010).

8 Cronin, C. N. & McIntire, W. S. pUCP-Nco and pUCP-Nde: *Escherichia–Pseudomonas* Shuttle Vectors for Recombinant Protein Expression in *Pseudomonas*. *Anal. Biochem.* **272**, 112-115, doi:http://dx.doi.org/10.1006/abio.1999.4160 (1999).

9 Guzman, L. M., Belin, D., Carson, M. J. & Beckwith, J. Tight regulation, modulation, and high-level expression by vectors containing the arabinose PBAD promoter. *J. Bacteriol.* **177**, 4121-4130 (1995).

10 Gödeke, J., Heun, M., Bubendorfer, S., Paul, K. & Thormann, K. M. Roles of two *Shewanella oneidensis* MR-1 extracellular endonucleases. *Appl. Environ. Microbiol.* **77**, 5342-5351, doi:10.1128/AEM.00643-11 (2011).

11 Kovach, M. E. *et al.* Four new derivatives of the broad-host-range cloning vector pBBR1MCS, carrying different antibiotic-resistance cassettes. *Gene* **166**, 175-176, doi:https://doi.org/10.1016/0378-1119(95)00584-1 (1995).
